# Montanide, Poly I:C and nanoparticle based vaccines promote differential suppressor and effector cell expansion: a study of induction of CD8 T cells to a minimal *Plasmodium berghei* epitope

**DOI:** 10.3389/fmicb.2015.00029

**Published:** 2015-02-06

**Authors:** Kirsty L. Wilson, Sue D. Xiang, Magdalena Plebanski

**Affiliations:** Department of Immunology, Central Clinical School, Faculty of Medicine, Nursing and Health Sciences, Monash University, Melbourne, VICAustralia

**Keywords:** malaria, adjuvant, nanoparticle, CD8 peptide, Treg, MDSC

## Abstract

The development of practical and flexible vaccines to target liver stage malaria parasites would benefit from an ability to induce high levels of CD8 T cells to minimal peptide epitopes. Herein we compare different adjuvant and carrier systems in a murine model for induction of interferon gamma (IFN-γ) producing CD8 T cells to the minimal immuno-dominant peptide epitope from the circumsporozoite protein (CSP) of *Plasmodium berghei,* pb9 (SYIPSAEKI, referred to as KI). Two pro-inflammatory adjuvants, Montanide and Poly I:C, and a non-classical, non-inflammatory nanoparticle based carrier (polystyrene nanoparticles, PSNPs), were compared side-by-side for their ability to induce potentially protective CD8 T cell responses after two immunizations. KI in Montanide (Montanide + KI) or covalently conjugated to PSNPs (PSNPs-KI) induced such high responses, whereas adjuvanting with Poly I:C or PSNPs without conjugation was ineffective. This result was consistent with an observed induction of an immunosuppressed environment by Poly I:C in the draining lymph node (dLN) 48 h post injection, which was reflected by increased frequencies of myeloid derived suppressor cells (MDSCs) and a proportion of inflammation reactive regulatory T cells (Treg) expressing the tumor necrosis factor receptor 2 (TNFR2), as well as decreased dendritic cell (DC) maturation. The other inflammatory adjuvant, Montanide, also promoted proportional increases in the TNFR2^+^ Treg subpopulation, but not MDSCs, in the dLN. By contrast, injection with non-inflammatory PSNPs did not cause these changes. Induction of high CD8 T cell responses, using minimal peptide epitopes, can be achieved by non-inflammatory carrier nanoparticles, which in contrast to some conventional inflammatory adjuvants, do not expand either MDSCs or inflammation reactive Tregs at the site of priming.

## INTRODUCTION

Malaria affects over 200 million people annually, resulting in over half a million deaths with most mortality coming from infections with *Plasmodium falciparum,* and developing a malaria vaccine has become a major global effort (Arama and Troye-Blomberg, 2014). The most advanced malaria vaccine development focuses on the pre-erythrocytic stage, at which sporozoite parasites enter the circulation after a mosquito bite and then rapidly enter and infect hepatocytes. CD8 T lymphocytes, particularly those capable of producing interferon gamma (IFN-γ), can mediate effective sterile liver-stage immunity (Schneider et al., 1999; Doolan and Martinez-Alier, 2006; Krzych et al., 2014). Developing a CD8 T cell inducing liver-stage vaccine would be beneficial to further avoid the clinical symptoms of malaria, such as fever, associated with subsequent blood stages of infection, as well as preventing transmission and the sexual development of parasites (Arama and Troye-Blomberg, 2014). Whole irradiated sporozoites are effective CD8 T cell inducing vaccines (Doolan and Martinez-Alier, 2006), and immunity to a dominant circumsporozoite protein (CSP) CD8 T cell epitope of *P. berghei*, named pb9 (sequence SYIPSAEKI), can mediate protection in murine animal models (Schneider et al., 1999).

Unfortunately, synthetic and recombinant vaccines have been less effective at inducing CD8 T cells, particularly in humans (Arama and Troye-Blomberg, 2014). The choice of adjuvant and the delivery system for the selected antigens will play a major role in the ability of vaccines to induce CD8 T cell immunity. Minimal CD8 T cell peptide epitopes offer production, stability, and flexibility advantages in vaccine formulation (Plebanski et al., 2006). Herein we compare side by side two adjuvants with proven capacity to promote CD8 T cell responses, Montanide (a water in oil emulsion) and Poly I:C (TLR3 agonist). Both have been used in various clinical trials as adjuvants in human vaccines against specific diseases (Aucouturier et al., 2002; Bonhoure and Gaucheron, 2006; Trumpfheller et al., 2008; Longhi et al., 2009; Mbow et al., 2010). Given cerebral malaria pathology is associated with inflammation (Postels and Birbeck, 2013), the use of novel nanovaccine technologies which induce CD8 T cell immunity without conventional pro-inflammatory signals also offers a conceptual advantage. Based on our previous studies, such inert nanoparticles coated with a target antigen of choice can promote high levels of immunity in the absence of inflammation or added extrinsic adjuvants, even to peptide based antigens (Fifis et al., 2004a,b; Xiang et al., 2013). Responses are as high as experimental gold standards for antibody production (e.g., Freunds adjuvant) and CD8 T cell induction [e.g., *ex-vivo* antigen pulsed dendritic cells (DCs)], and better than a range of conventional inflammatory experimental adjuvants (Fifis et al., 2004a).

The size of the nanoparticle is a key factor, with even small deviations away from the optimal size range of 40–50 nm causing major decreases in immunogenicity (Fifis et al., 2004a; Mottram et al., 2007). We herein compared Montanide and Poly I:C, representing two pro-inflammatory adjuvants, against such nanoparticle based vaccines for delivery of the minimal pb9 CD8 T cell epitope. Moreover, we speculated that inflammatory responses during the priming phase of immunity could further result in the activation of the immune-suppressive mechanisms that arise to control such inflammation, but may interfere with efficient CD8 T cell stimulation. In this context, it is known that enhancing cross-presenting DC frequency and function, and preventing myeloid derived suppressor cells (MDSCs) accumulation promotes antigen specific immune responses (Ohkusu-Tsukada et al., 2011). It has also been suggested that Poly I:C is capable of increasing antigen specific effector T cells over regulatory T cells (Treg), enhancing immunity (Perret et al., 2013). Hence, as well as comparing the magnitude of the CD8 T cell responses induced by the different adjuvants, this study evaluates the ability of Montanide, Poly I:C, and nanoparticles to promote the induction of inflammation reactive Tregs and the expansion of MDSCs, compared to effector T cells and stimulatory antigen presenting types such as DCs.

## MATERIALS AND METHODS

### MICE

Six to eight weeks old BALB/c mice were purchased from Monash Animal Services (MAS) Melbourne, VIC, Australia. The studies presented here were approved by the Alfred Medical Research and Education Precinct (AMREP) Animal Ethics Committee, Melbourne, VIC, Australia.

### NANOVACCINE FORMULATIONS

Conjugation of malaria peptide antigens to nanoparticles was based on the previous described method (Xiang et al., 2013) with a slight modification. Briefly, carboxylated polystyrene nanoparticles (PSNPs; Polysciences Inc, Warrington, PA, USA) of 40 nm (∼40–50 nm) at a final of 1% solids were activated in a mixture containing 2-N-Morpholino-ethanesulfonic acid (MES; 50 mM final, pH = 7), and 1-ethyl-3-(3-dimethylaminopropryl) carbodiimide hydrochloride (EDC; 4 mg/ml final). Malaria peptide SYIPSAEKI (KI; Mimotopes, Melbourne, VIC, Australia; 1 mg/ml final) was also added to the conjugation mix and together incubated on a rotary wheel at room temperature for approximately 4 h. Following antigen incubation, the conjugation reactions were then quenched by adding excess glycine (7 mg/ml final) and further incubated for 30 min. The free, unconjugated peptide antigens, and other excess conjugation agents, were removed by dialysis (10–14 kDa molecular weight cut-off (MWCO) membrane; Viskase, Darien, IL, USA) against phosphate buffered solution (PBS, pH = 7.2) at 4°C overnight. Conjugation efficiency and final sizes of the nanovaccine formulation (e.g., PSNPs-KI) were determined by Bicinchoninic acid assay (BCA; Thermo Fisher Scientific, Rockford, IL, USA) and dynamic light scattering instruments (Zetasizer; Malvern Instruments, Worcestershire, UK), respectively, following the manufacture’s instruction.

### OTHER VACCINE ADJUVANT AND IMMUNIZATIONS

Other vaccine adjuvants such as Montanide ISA 720 (70% v/v final, Tall Bennett Group, USA), Polyinosinic–polycytidylic acid sodium salt (Poly I:C; 25 μg/mouse final, Sigma Aldrich, St. Louis, MO, USA) were also used in this study. The adjuvant effect of these vaccine formulations were tested *in vivo* by immunization of mice and measuring for IFN-γ production by ELISpot assay (Xiang et al., 2006). Briefly, adjuvant mixed KI formulations (e.g., KI + Montanide; KI + PolyI:C; KI + PSNPs) at the desired final concentrations (∼25 μg KI/mouse) and nanoparticle conjugated KI (PSNPs-KI at 1% solid of PSNPs) formulations were injected into mice intradermally (i.d.) at the base of the tail. 14 days after the last immunization, mice were sacrificed and splenocytes were isolated and assayed for IFN-γ production via ELISpot assay.

### ELISPOT ASSAY

Antigen specific CD8 T cell responses were evaluated by IFN-γ ELISpot assay (Xiang et al., 2006). Briefly, 96 well multiscreen filter plates (MSIP plates, Millipore, Billerica, MA, USA) were coated with 5 μg/ml final (100 μl/well) of anti-mouse IFN-γ (AN18, MABTech, Stockholm, Sweden) in PBS, and incubated overnight at 4°C. Following overnight incubation, the wells were washed and blocked with RPMI 1640 (Gibco, Life Technologies, Carlsbad, CA, USA), supplemented with 10% fetal calf serum (FCS; Gibco, Life Technologies), 100 units/ml penicillin, 100 μg/ml streptomycin, 2 mM L-glutamine, 1 M Hepes, and 0.1 mM 2-mercaptoethanol, for a minimum of 1 h. Splenocytes from mice (immunized with or without vaccine formulations as listed above) were added in triplicate wells (1 × 10^7^ cells/ml, 50 μl/well), along with the recall antigen (peptide SYIPSAEKI at different doses, 50 μl/well). Media alone control, or concanavalin A (ConA; Amersham Biosciences, Uppsala, Sweden; final 1 μg/ml) were also used, as negative or positive controls, respectively. All mice produced high levels of IFN-γ in response to ConA, with SFU often above the threshold for accurate counting (data not shown), indicating adequate cell viability and functionality. Cells with antigens were incubated in a 37°C incubator filled with 6% CO_2_ for a minimum of 16 h. Plates were then washed five times in PBS, biotinylated detection antibody anti-IFN-γ (R4-6A2-Biotin, MABTech; 1 μg/ml in PBS 0.5% FCS, 100 μl/well) was added and followed by further incubation at room temperature for 2 h. Plates were then washed again, as above, and streptavidin-alkaline phosphatase enzyme conjugate (ALP; 1 μg/ml in PBS 0.5% FCS, 100 μl/well; MABTech) was added, followed by a further 1.5 h incubation at room temperature. After a final wash in PBS and followed by water, the spots were developed using a colorimetric AP kit (Bio-Rad, Philadelphia, PA, USA) following the manufacturer’s instructions. Spots were counted by an AID ELISpot Reader System (Autoimmun Diagnostika GmbH, Germany).

### FLOW CYTOMETRY

For phenotypic analysis of cells by flow cytometry, inguinal lymph node cells were isolated 48 h after immunization with the adjuvants alone. 2 × 10^6^ cells/sample were stained for 15 min at room temperature with 30 μl of antibody cocktails, including antibodies with different fluorochromes at different concentrations based on prior optimizations. Antibodies used in the present study include; anti-CD11c V450 (HL3), anti-CD11b PeCy7 (M1/70), anti-Gr-1 (Ly6C and Ly6G) PerCP Cy5.5 (RB6-8C5), anti-CD3 AF700 (500A2), anti-CD4 BV605 (RM4-5, Biolegend, San Diego, CA, USA), anti-CD8 BV650 (53-6.7, Biolegend), anti-CD25 PeCy7 (PC61), anti-FoxP3 APC (MF23), and anti-CD120b (TNFR2) PE (TR75-89). All antibodies were from BD Biosciences (NJ, USA) except where specifically indicated. Following incubation, cells were washed with 100 μl PBS/2% FCS (FACS buffer). Stained cells were fixed with 1% (v/v) paraformaldehyde (PFA, Sigma Aldrich) and acquired using an LSRII flow cytometer (BD Biosciences) located at the AMREP Flow Cytometry Core Facility (Melbourne, VIC, Australia). Data was analyzed using FlowJo software (version10, Treestar, USA).

### STATISTICAL ANALYSIS

Statistical analysis was done by ANOVA analysis, with *post hoc* Tukeys multiple comparison tests or Fisher’s LSD test, or unpaired *t*-tests, using Graphpad Prism software (version 6, San Diego, CA, USA). Statistical significance was determined as *p* < 0.05. Group sizes are indicated in the figure legends. All values are expressed as mean ± SD.

## RESULTS

### PEPTIDE COVALENTLY BOUND TO, BUT NOT MIXED WITH, PSNPs INDUCES CD8 T CELLS

**FIGURE 1:**
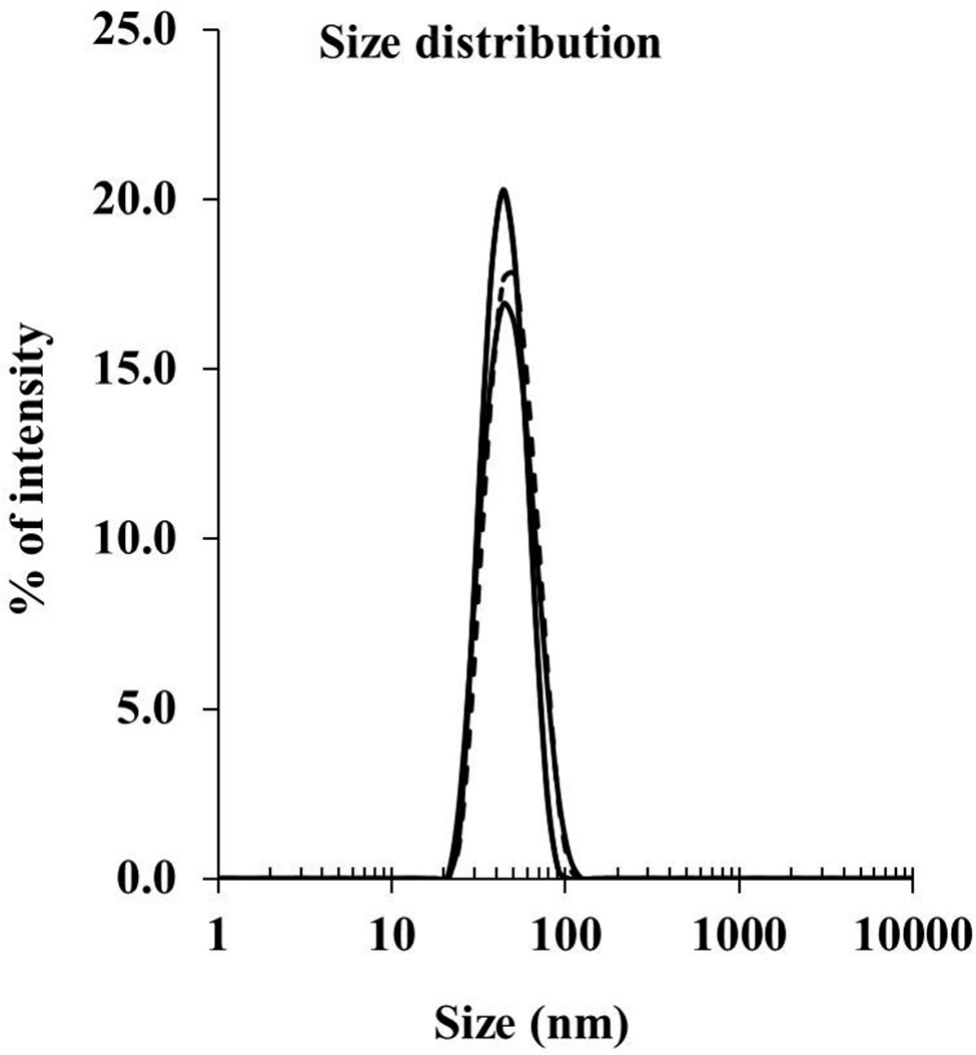
**Size distribution for PSNPs-KI formulations.** SYIPSAEKI peptides were covalently conjugated to PSNPs, and the final sizes were measured by dynamic light scattering instruments (Zetasizer).

Peptide delivery by nanoparticles (either mixed or conjugated) was compared for immunogenicity *in vivo* using BALB/c mice. To generate the conjugated nanovaccine, the immune-dominant CD8 T cell peptide epitope of the CSP protein, SYIPSAEKI (KI), from *P. berghei* was covalently attached to carboxylated polystyrene nanoparticles (PSNPs, 40–50 nm) using an optimized covalent conjugation protocol as previously described (Xiang et al., 2013). As shown in **Table 1**, the average size of the PSNPs-KI formulation was 47.97 ± 2.64 nm, and the polydispersity index (PdI) was very low (0.07 ± 0.03), indicating the successful formulation of a uniformly dispersed nanoparticle formulation with a narrow size distribution range (**Figure 1**). The antigen loading was 0.32 ± 0.09 mg/ml, which represented 1032.6 ± 147.8 peptide molecules per particle (**Table 1**). The number of peptide molecules per particle was comparable to previous studies with a model peptide antigen, SIINFEKL, where potent responses were observed at that loading (Xiang et al., 2013).

**Table 1:** Characterization of SYIPSAEKI conjugation to PSNPs (PSNPs-KI) for size, polydispersity, and peptide loading*.

Formulation	Size (nm)	Polydispersity index (PdI)	Peptide molecules per particle
PSNPs-KI	47.97 ± 2.64	0.07 ± 0.03	1032.6 ± 147.8

*Data presented as mean ± SD, *n* = 4 repeated conjugations.

**FIGURE 2:**
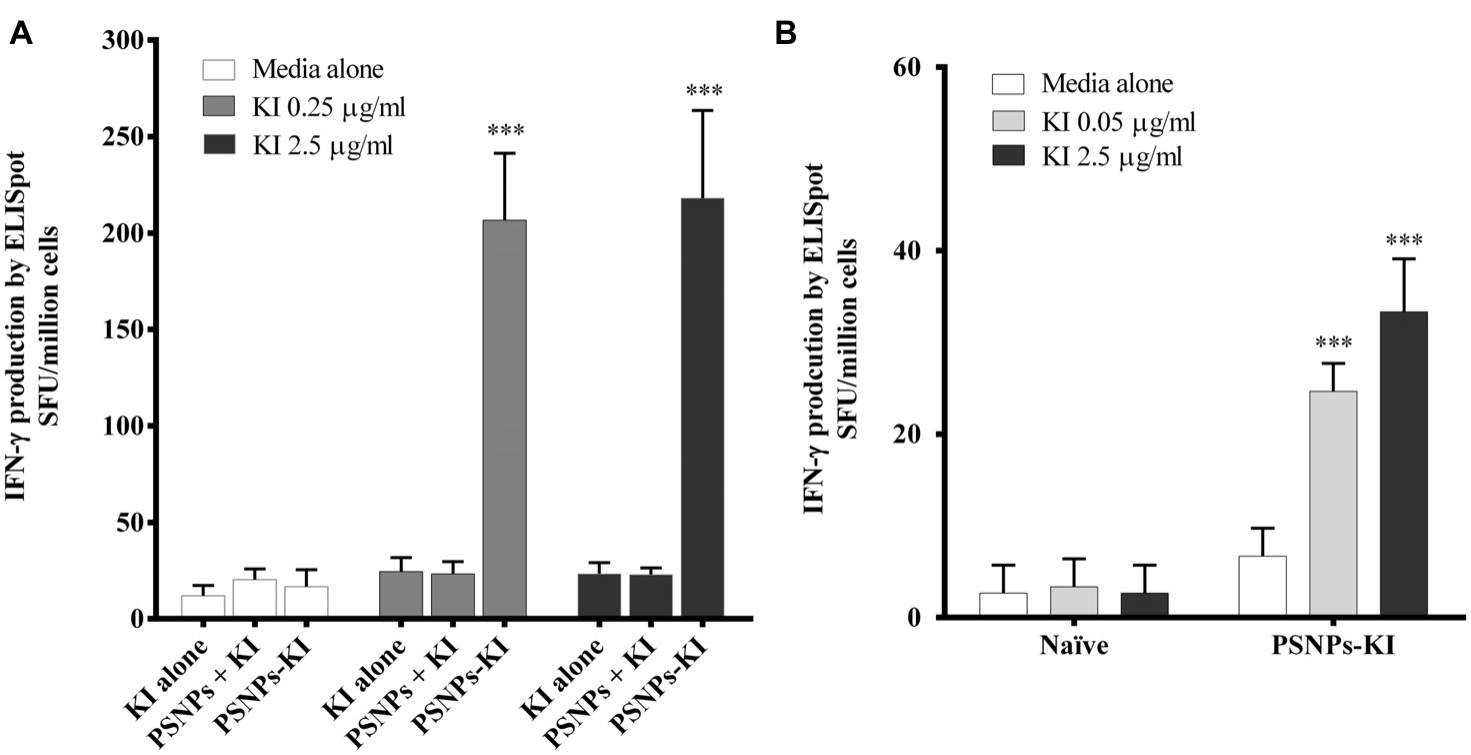
**Antigen specific CD8 T cell responses induced by SYIPSAEKI peptide vaccines in combination with PSNPs.** Mice (BALB/c) were immunized with SYIPSAEKI peptide mixed with or conjugated to PSNPs intradermally at the base of tail. 14 days after the last immunization, spleen cells were collected and assessed for IFN-γ production by ELISpot assay. **(A)** KI peptides conjugated to, but not mixed with, PSNPs induced high levels of KI specific CD8 T cell responses after two immunizations (2 weeks apart). Data presented as mean ± SD of SFU/million cells from each group (*n* = 3 mice/group). **(B)** Immunogenicity of PSNPs-KI formulation after one immunization (*n* = 4 mice per group). Data presented as mean ± SD of SFU/million cells (pooled for each group) from the triplicated wells in ELISpot assay. Statistical analysis was performed via ANOVA, ***p ≤ 0.001.

Mice were immunized twice, 14 days apart, with SYIPSAEKI peptide either alone (KI alone), mixed with the PSNPs (PSNPs + KI), or covalently conjugated to the PSNPs (PSNPs-KI) at the dosage of ∼25 μg of KI/mouse/injection. As results show in **Figure 2A**, neither the “KI alone” nor the “PSNPs + KI” treatment groups showed induction of KI specific CD8 T cell responses, assessed by IFN-γ ELISpot after two immunizations. However, when mice were immunized with KI conjugated to PSNPs (PSNPs-KI), significant (*p* < 0.001) levels of KI specific IFN-γ producing CD8 T cells were induced, even at the smallest amount of recall antigen concentration (0.25 μg/ml). Increasing the recall antigen concentration did not further enhance the overall antigen specific IFN-γ responses, suggesting the recall of high affinity T cells. These results also show that this specific malaria antigen peptide needs to be covalently conjugated to its carrier nanoparticles to induce potent immune responses. Moreover, it shows that this system can utilize minimal CD8 T cell epitopes, without added CD4 T cell epitopes, and still induce levels of immune responses previously associated with powerful ‘Prime-boost’ immunization modalities and sterile protection against sporozoite challenge (Plebanski et al., 1998).

Given the PSNPs-KI formulation induced potent immune responses with two immunizations; we further tested formulation potency in a single dose immunization regime. As shown in **Figure 2B**, after one immunization, PSNPs-KI formulations induced good antigen specific CD8 T cell responses, significantly higher than naïve controls (*p* < 0.001, **Figure 2B**). Recall T cells were elicited in ELISpot at both 2.5 and 0.05 μg/ml, suggesting high affinity T cells were induced already in the initial priming phase.

### PSNPs-KI AND MONTANIDE INDUCE THE HIGHEST CD8 T CELL RESPONSES

**FIGURE 3:**
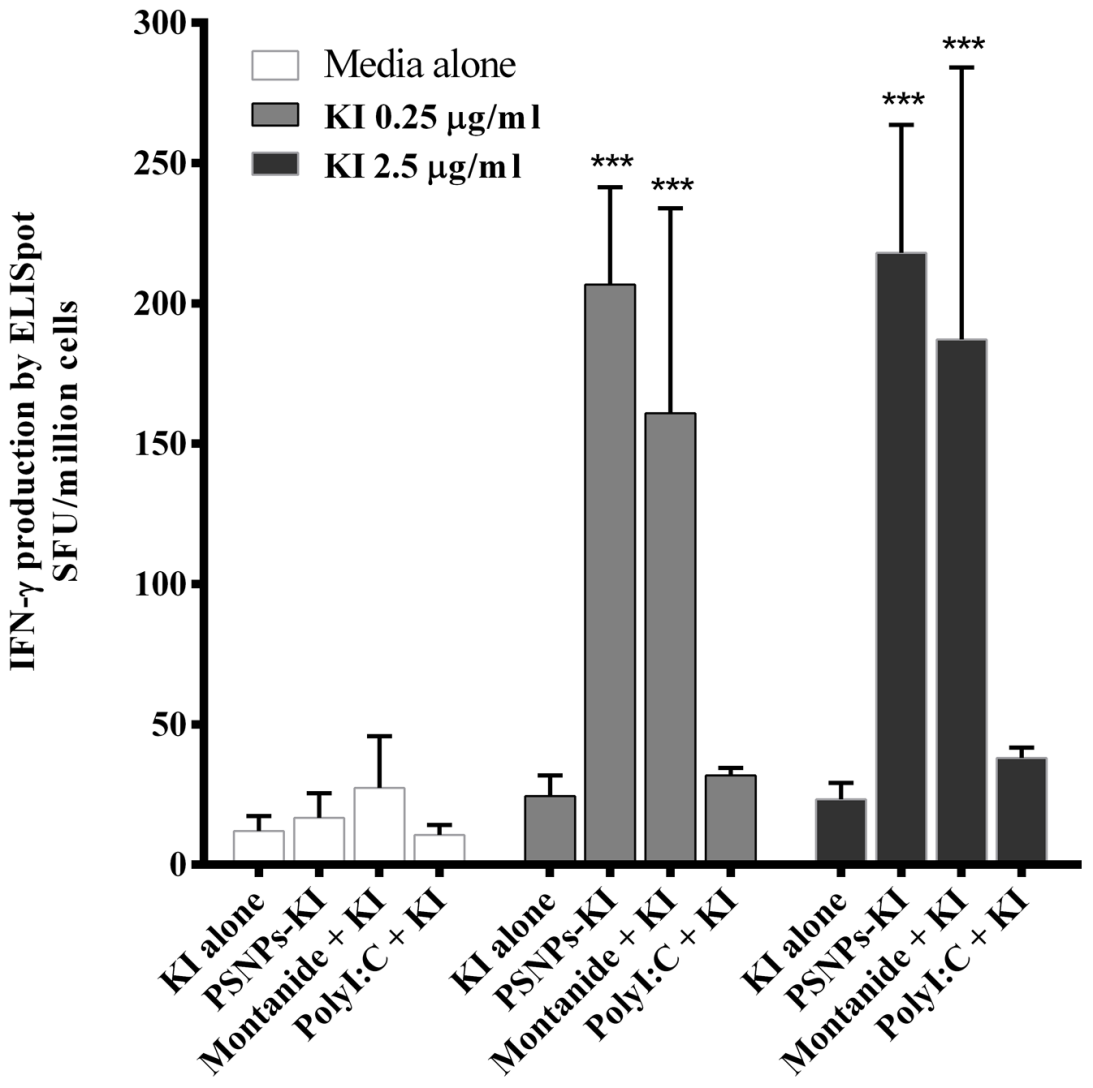
**Induction of SYIPSAEKI specific CD8 T cells by different adjuvants.** BALB/c mice were immunized twice, intradermally, 2 weeks apart, with SYIPSAEKI peptides incorporated with respective adjuvants. 14 days after the last immunization, spleen cells were collected and assessed for IFN-γ production by ELISpot assay. Data presented as mean ± SD of SFU/million cells from each group (*n* = 3 mice/group). Statistical analysis was performed via ANOVA, ***p ≤ 0.001.

To benchmark the immunogenicity of PSNPs-KI compared to other types of conventionally adjuvanted experimental formulations capable of inducing CD8 T cell responses, we further tested Montanide and Poly I:C with KI side by side with PSNPs-KI. Strong and comparable KI specific CD8 T cell responses were detected in mice immunized with “PSNPs-KI” and “Montanide + KI” formulations (**Figure 3**). The magnitude of the KI specific IFN-γ production by both these formulations was significantly higher (*p* < 0.001) than that from mice immunized with KI alone or “Poly I:C + KI” (**Figure 3**). Despite the literature indicating that Poly I:C is a potent CD8 T cell response inducer (Nordly et al., 2011), Poly I:C mixed with KI formulation didn’t promote the induction of IFN-γ producing CD8 T cells above that induced with peptide alone, after two immunizations.

### POLY I:C, BUT NOT PSNPs OR MONTANIDE, IS ASSOCIATED WITH A LACK OF DC MATURATION 48 H POST INJECTION WITH THE ADJUVANT ALONE

**FIGURE 4:**
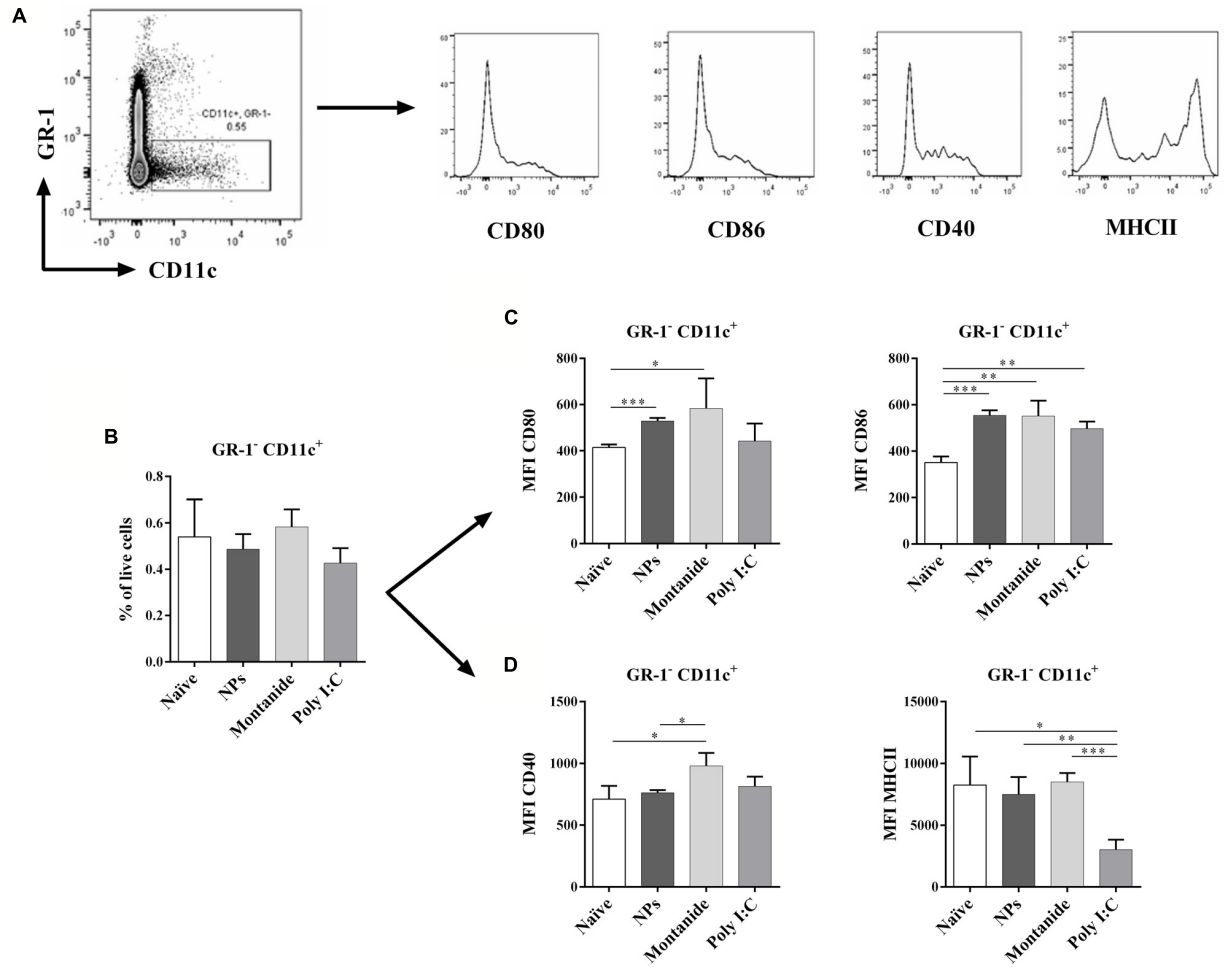
**Dendritic cell activation in dLNs after injection with PSNPs, Montanide, and Poly I:C.** Mice (BALB/c) were injected once intradermally at the base of tail with the different adjuvants alone. 48 h after injection, mice were sacrificed, local (inguinal) dLNs were harvested and the levels of CD11c^+^ DCs and various activation markers were assessed by flow cytometry. **(A)** gating strategy; **(B)** frequency of GR-1^-^CD11c^+^ cells; **(C)** Mean fluorescent intensity (MFI) of CD80 and CD86 on GR-1^-^CD11c^+^ cells; **(D)** MFI of CD40 and MHCII on GR-1^-^CD11c^+^ cells. Data presented as mean ± SD of MFI for each group of treatment (*n* = 3 mice/group). Statistical analysis was performed via *t*-tests, *p ≤ 0.05, **p ≤ 0.01, ***p ≤ 0.001.

To further understand how the potent CD8 T cell responses could be induced by a single CD8 T cell epitope when conjugated to PSNPs in the absence of a CD4 T cell helper epitope, as well as to compare the non-specific action mode of other adjuvants alone in the induction of cell activation, we investigated the level of DC activation in the local draining lymph node (dLN). This was done by assessing the expression levels of MHCII, CD40, CD80, and CD86 on the CD11c^+^ DCs, from the inguinal lymph node, after the injection of PSNPs, Montanide or Poly I:C *in vivo,* in the absence of antigen. We hypothesized that there would be efficient CD86 induction on DC, making them highly capable of activating CD8 T cells (Clarke, 2000; Steinman et al., 2003; Maroof et al., 2009). The critical time-period for CD8 T cell expansion is between 48 and 72 h post priming, a period of repeated transient contact between T cells and DC (Henrickson et al., 2008). Increases in suppressor cells would be expected to follow initial inflammation induced by adjuvants, which usually peaks at 12–24 h post administration. Therefore, between 24 and 72 h immunosuppressive mechanisms would be expected to come into play. We assessed DC frequency and expression of co-stimulatory molecules in adjuvant dLN 48 h after injection with the adjuvants alone. Results in **Figure 4A** (gating strategy) and **Figure 4B** show that the overall frequency of DCs (Gr-1^-^ CD11c^+^ cells) remained the same in the dLN 48 h post injection with all three types of carrier/adjuvants. There was a significant increase in the expression of CD80 in the dLN DCs after treatment by both PSNPs and Montanide (*p* < 0.001 and *p* < 0.05, respectively, **Figure 4C**). Furthermore, there was a significant increase in expression levels of CD86 on CD11c^+^ cells for all adjuvants tested (*p* < 0.001 compared to PSNPs, and *p* < 0.01 compared to Montanide and Poly I:C treatment, **Figure 4C**), implying DCs were potentially being activated even in the absence of a CD4 T cell helper epitope. CD11c^+^ DCs in the Montanide group further showed an increase in the expression of CD40, compared to the naïve and PSNPs groups (*p* < 0.05, **Figure 4D**). However, surprisingly, DCs in the Poly I:C treated group showed significantly lower levels of expression of MHCII compared to all other treatment groups (*p* < 0.05 compared to naïve, *p* < 0.01 compared to PSNPs and *p* < 0.001 compared to Montanide treatment, **Figure 4D**), suggesting these DCs were at a different state of maturation, and/or activation, upon treatment with Poly I:C.

### PSNPs AND MONTANIDE INDUCE AN ENVIRONMENT THAT ENCOURAGES STIMULATORY DCs, WHEREAS POLY I:C PROMOTES SUPPRESSIVE MDSCs 48 H POST INJECTION WITH THE ADJUVANTS ALONE

**FIGURE 5:**
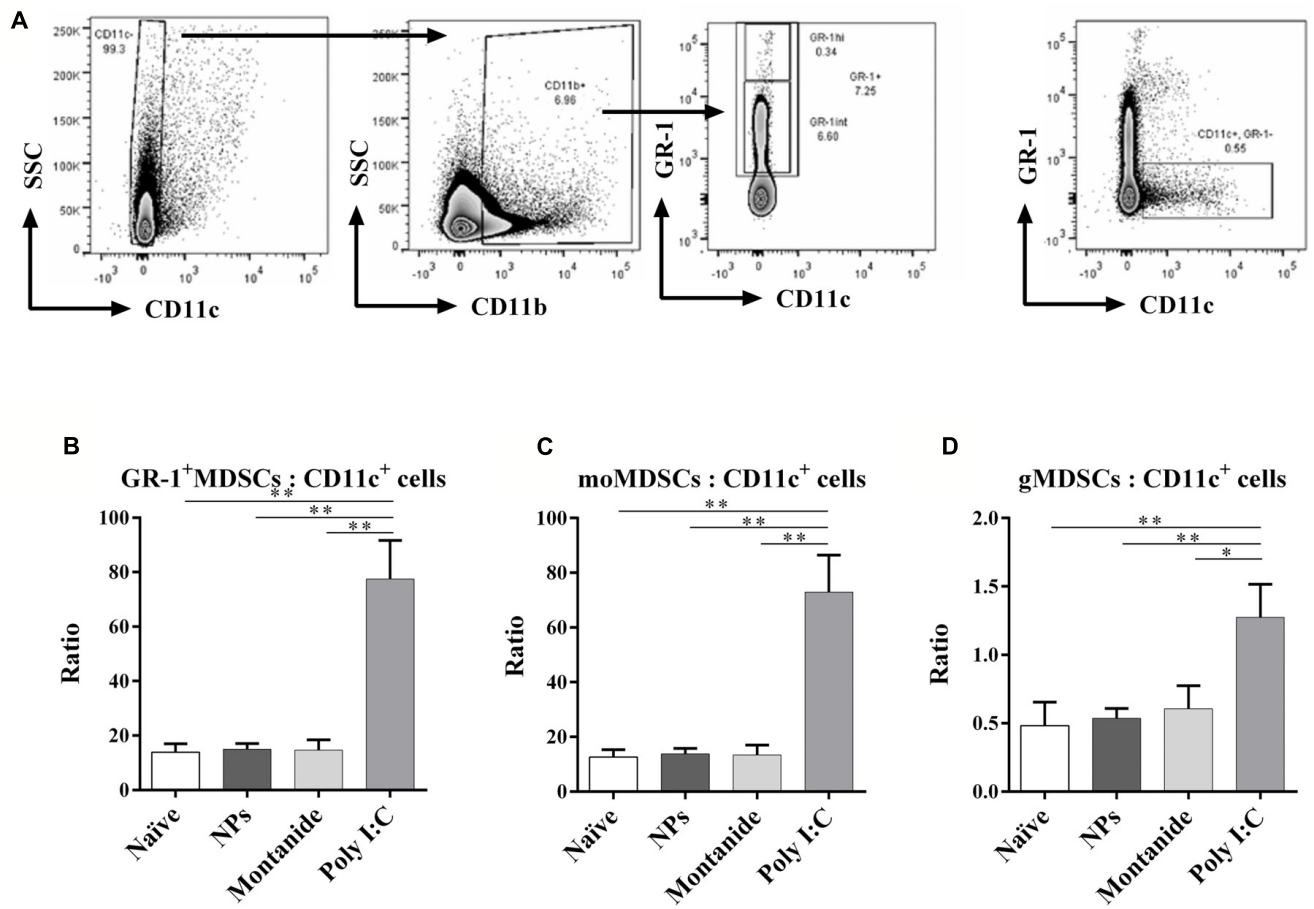
**Differential expression of GR-1^**+**^ MDSCs and DCs in the local dLN.** Mice (BALB/c) were injected once intradermally at the base of the tail with the different adjuvants alone. 48 h after injection, mice were sacrificed, local (inguinal) dLNs were harvested and levels of GR-1^+^ MDSCs and DCs, and their ratios, were assessed by flow cytometry. **(A)** gating strategy; **(B)** ratio of GR-1^+^ MDSCs: CD11c^+^ cells; **(C)** ratio of moMDSCs: CD11c^+^ cells; **(D)** ratio of gMDSCs: CD11c^+^ cells. Data presented as mean ± SD of ratio for each group of treatment (*n* = 3 mice/group). Statistical analysis was performed via *t*-tests, *p ≤ 0.05, **p ≤ 0.01.

The balance between DCs and MDSCs in the priming lymph node would be predicted to influence the level of immunity subsequently induced by vaccines. We further assessed the MDSC and CD11c^+^ DC populations in the dLN 48 h after injection (**Figure 5A**). Whilst PSNPs and Montanide maintained a normal MDSC to DC ratio in the dLN (**Figure 5B**), surprisingly, Poly I:C promoted a significantly higher ratio of MDSCs to DCs, compared to all other groups (*p* < 0.01, **Figure 5B**). Further analysis of the subsets within MDSCs, based on their level of Gr-1 expression, showed that Poly I:C significantly increased the ratio of MDSCs expressing intermediate levels of Gr-1 (monocytic or suppressive MDSC, moMDSC; Kong et al., 2013) over DC, moMDSC/CD11c, when compared to naïve, Montanide or PSNPs treated groups (*p* < 0.01, **Figure 5C**). There was also a significant increase in the ratio of MDSC expressing high levels of Gr-1 (granulocytic MDSC, gMDSC; Kong et al., 2013) to DC (gMDSCs/CD11c) for all treatments (*p* < 0.01 compared to naïve and PSNPs, and *p* < 0.05 compared to Montanide treatment, **Figure 5D**), however, the magnitude of this increase was not as high as the increased ratio of moMDSC/CD11c cells. Hence Poly I:C induced an environment abundant in suppressive (monocytic) phenotype MDSC in the dLN within a short time frame, whereas PSNPs and Montanide did not promote increases in such MDSCs.

### POLY I:C AND MONTANIDE PROMOTE THE INDUCTION OF TNFR2^**+**^ TREG CELLS

**FIGURE 6:**
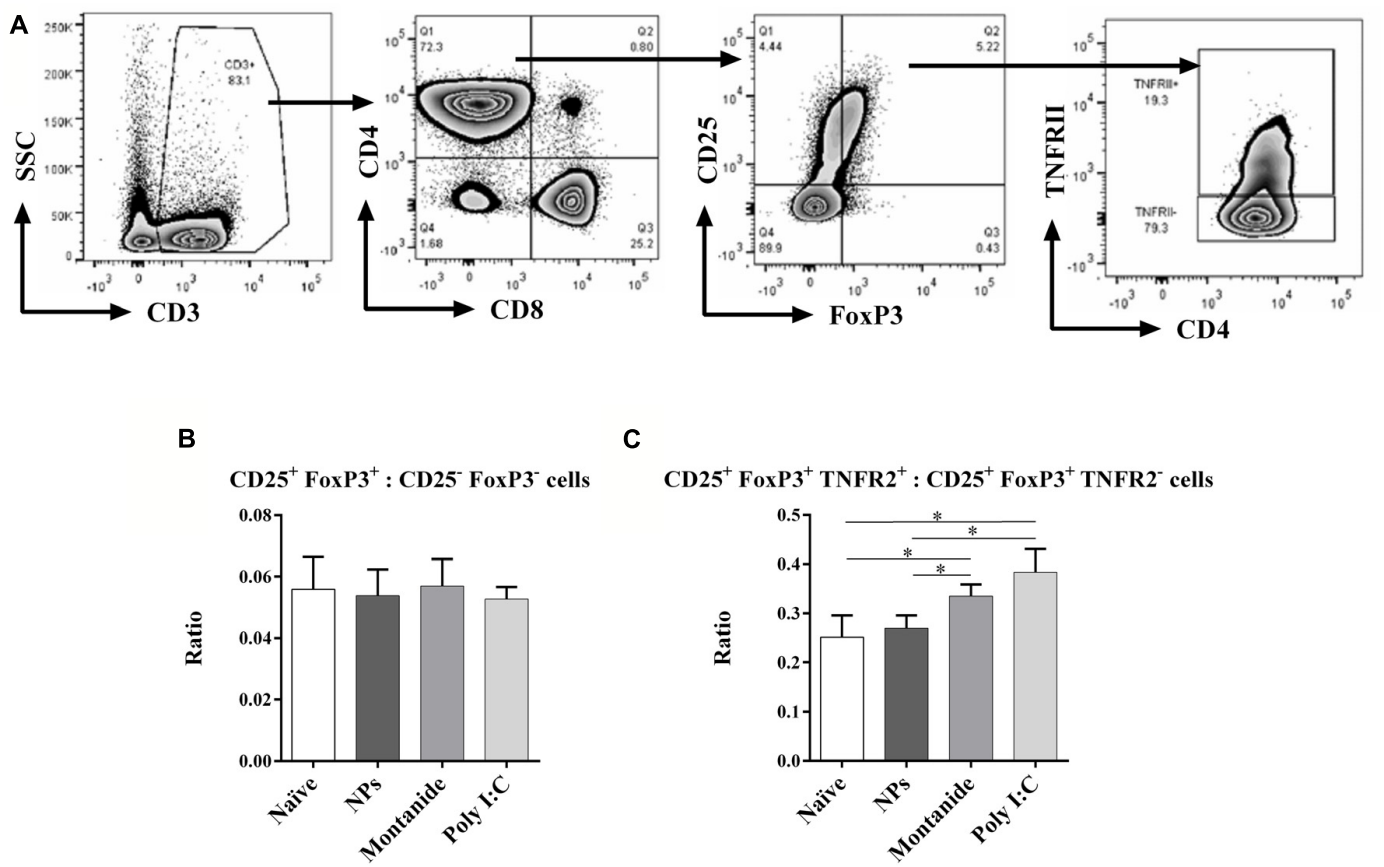
**Differential expression of CD4^**+**^ Treg and effector T cells in the local dLN.** Mice (BALB/c) were injected once intradermally at the base of tail with the different adjuvants alone. 48 h after injection, mice were sacrificed, local (inguinal) dLNs were harvested and the levels of CD4^+^ Treg and effector T cells, and their ratios, were assessed by flow cytometry. **(A)** gating strategy; **(B)** ratio of FoxP3^+^CD25^+^: FoxP3^-^CD25^-^ cells; **(C)** ratio of FoxP3^+^CD25^+^TNFR2^+^: FoxP3^+^CD25^+^TNFR2^-^ cells. Data presented as mean ± SD of ratio for each group of treatment (*n* = 3 mice/group). Statistical analysis was performed via *t*-tests, *p ≤ 0.05.

Inflammatory, tumor necrosis factor (TNF) inducing, adjuvants such as Montanide and Poly I:C, have the potential to stabilize FoxP3 expression on Treg that express the TNF receptor 2 (TNFR2; Chen and Oppenheim, 2011). TNFR2 has also previously been found to identify the most highly active and immunosuppressive Treg subset (Govindaraj et al., 2014). We speculated that pro-inflammatory adjuvants could therefore increase the Treg to T effector ratio in the dLN, and if this occurred during the T cell priming phase, it could potentially interfere with effective CD8 T cell induction. We further analyzed the Treg and T effector cells in the dLN (**Figure 6A**), and found that whilst there was no overall increase in total Treg to T effector cell ratio (CD25^+^FoxP3^+^ to CD25^-^FoxP3^-^ cells; **Figure 6B**), Poly I:C and Montanide significantly increased the frequency of TNFR2^+^ Treg (FoxP3^+^CD25^+^TNFR2^+^ cells) compared to TNFR2^-^ Treg (FoxP3^+^CD25^+^TNFR2^-^ cells) in the dLN 48 h post injection, compared to the naïve and PSNPs groups (*p* < 0.05, **Figure 6C**). PSNPs maintained the balance of TNFR2^+^ to TNFR2^-^ Treg subpopulations.

## DISCUSSION

The side-by-side comparison of three different adjuvant systems for the induction of highly responsive CD8 T cells to a minimal peptide epitope antigen from CSP of *P. berghei* demonstrated that: (1) Non-inflammatory and inflammatory vaccines can elicit similarly high levels of immune responses, (2) Non-inflammatory nanovaccines require the minimal CD8 T cell epitope peptide to be covalently attached to the nanoparticle carrier, suggesting peptide delivery *in vivo* is key for antigenic stimulation, (3) Vaccines can prime high levels of CD8 T cells by delivering the minimal CD8 T cell epitope, without helper CD4 epitopes, (4) Inflammatory, but not non-inflammatory, adjuvants result in the induction of TNFR2^+^ Treg in dLNs during a timeframe consistent with the priming of an immune response, and (5) Together with the induction of enhanced numbers of suppressor moMDSC, such findings may explain the particularly poor capacity of Poly I:C to induce CD8 T cell immune responses.

Both “Montanide + KI” and “PSNPs-KI” formulations induced a similar magnitude of response after two immunizations, reaching the minimum threshold IFN-γ production levels determined to be required for sterile protection in the *P. berghei* challenge model (Plebanski et al., 1998). Given a threshold of 100 spots was required to start seeing sterile protection in about 74% of animals in previous studies (Plebanski et al., 1998), it is likely that the 200 spots achieved by the nanovaccines would also be protective, although it will be important to confirm this formally. The potentially protective IFN-γ levels produced in this study merit additional validation in further direct challenge studies. There may be additional advantages in using a non-inflammatory nanoparticle approach over Montanide. Montanide is a viscous combination of adjuvant with peptide, creating a depot at the injection site with the antigen, associated with some pain and local inflammation. As well as increasing compliance with vaccination, the use of a non-inflammatory adjuvant system that substantially drains to the lymph nodes, may, in the case of immunization of individuals in malaria endemic areas, help minimize the risk of triggering inflammatory feedback loops, such as those associated with cerebral malaria. Previous studies have shown nanoparticle based vaccines do not need to engage conventional inflammatory pathways to induce adaptive immunity (Karlson Tde et al., 2013; Xiang et al., 2013), and act by selectively targeting DCs, particularly CD8^+^ DCs, directly in the local lymph nodes (Fifis et al., 2004a; Mottram et al., 2007; Xiang et al., 2013), as well as by promoting uptake by DC in the periphery followed by subsequent migration via the afferent lymphatics (Gamvrellis et al., 2013). The critical factor identified that promotes CD8^+^ DCs targeting was found to be particle size (40–50 nm; Fifis et al., 2004a; Mottram et al., 2007). The fact that mixed-in nanoparticles in this study did not act as conventional adjuvants, and hence the carrier activity of the nanoparticles was sufficient and necessary to induce high levels of immune responses, predicts that nanoparticle carriers of the correct size to target CD8^+^ DCs *in vivo* (made of non-inflammatory materials) would also be capable of inducing high levels of immunity. Given the explosion in nanomaterials and delivery systems, this appears to be a promising and timely finding.

It was surprising to find that the “Poly I:C + KI” formulation was unable to induce similarly high CD8 T cell responses when compared side-by-side with the “Montanide + KI” and “PSNPs-KI” formulations. This could be mechanistically explained by the new finding that Poly I:C promotes dramatic increases in the ratio of MDSCs to DCs, including moMDSCs, in the LNs draining the injection site, within a timeframe capable of interfering with local CD8 T cell priming. Moreover, whereas the frequency of DCs remained the same in the dLN 48 h post injection with either PSNPs, Montanide or Poly I:C, there were significantly lower levels of MHCII expression on DCs treated with Poly I:C. Down-regulation of some activation markers on DCs has been associated in the literature with increases in suppressor MDSC frequencies and their subsequent apoptosis (Shen et al., 2014). MDSCs can suppress effector T cell responses directly, or by promoting the expansion of Tregs in the presence of IFN-γ (Kong et al., 2013).

Together our results suggest that non-inflammatory nanoparticles 40–50 nm or Montanide can be used to induce potent CD8 T cell responses, even when used with purely a minimal CD8 T cell peptide epitope. Generally, the results herein also suggest a new paradigm for highly immunogenic vaccines, which could instead of delivering pro-inflammatory danger signals, be designed to ‘keep under the radar’ to deliver antigen to cross-priming CD8^+^ DCs whilst avoiding the expansion of some key immunosuppressive and inflammation reactive cell populations.

## Conflict of Interest Statement

The authors declare that the research was conducted in the absence of any commercial or financial relationships that could be construed as a potential conflict of interest.
